# Perceived Threat of the Coronavirus and the Role of Trust in Safeguards: A Case Study in Slovakia

**DOI:** 10.3389/fpsyg.2020.554160

**Published:** 2020-11-30

**Authors:** Martin Kanovsky, Júlia Halamová

**Affiliations:** ^1^Institute of Social Anthropology, Faculty of Social and Economic Sciences, Comenius University in Bratislava, Bratislava, Slovakia; ^2^Institute of Applied Psychology, Faculty of Social and Economic Sciences, Comenius University in Bratislava, Bratislava, Slovakia

**Keywords:** coronavirus, confidence, risk, safeguards, public health

## Abstract

In this exploratory research study, we developed an instrument to investigate people’s confidence in safeguarding measures [Confidence in Safeguards Scale (CSS)] and we adapted an instrument measuring perceived risk of coronavirus [perceived risk of coronavirus scale (PRCS)] that was originally based on a perceived risk of HIV measure. We then explored the effect of public confidence in safeguarding measures designed to halt the spread of the coronavirus on perceived risk, controlling for related covariates. The sample consisted of *N* = 565 respondents; 119 were males (21.1%) and 446 were females (78.9%). Mean age was 35.42 (SD = 13.11), range was 18–77 years. We used convenience sampling to gather the data at the end of March 2020 via social media in Slovakia. The CSS showed good reliability levels and a three-factor structure: Confidence in Institutions, Confidence in Personal and Family Behaviors, and Confidence in Others’ Behaviors. The PRCS showed good reliability levels and a two-factor structure: Fear of Contraction and Perceived Likelihood of Contraction. Participants with higher levels of Confidence in Others’ Behaviors perceived the spread of the coronavirus to be less threatening, both cognitively (less perceived likelihood of contraction) and affectively (less fear of contraction). This finding could be used when designing public health policy and emergency communication. Enhancing confidence in others’ behaviors could encourage individual responsibility, social responsibility, and solidarity through social bonds extending beyond the family. In future research we plan to replicate the data collection using the same instruments in different countries so the results are comparable across cultures and can be used to improve emergency communication.

## Introduction

The coronavirus outbreak has triggered an unprecedented crisis. No other contagious epidemic (MERS, Ebola, swine flu) has led to such large safeguarding measures in Slovakia. In situations such as this, it is important to gather information on public perceptions of risk, the preparedness of people and institutions, protective behaviors, and trust and confidence in the safeguards so adequate responses can be taken. The public is exposed to a constant flow of information from news channels and social media, leading to information overload during the public health emergency. There is no doubt the spread of coronavirus is perceived as a threat and a risk. Public and private institutions have implemented many safeguarding measures to tackle the situation. Public confidence in these safeguarding measures is crucial and should help alleviate the perceived risk. However, careful consideration is required: it is crucial to avoid panic, but the public response should not be light-hearted either. The most desirable outcome is a careful balance of worry and just enough fear to change peoples’ behavior without the destructive effects of public panic. Risk communication in a public health emergency (Glik, 2007) should be designed whilst bearing in mind its effect on public trust. Carpenter (2010) demonstrated in a meta-analysis that if people perceive a negative health outcome to be severe and think they are susceptible, they are more inclined to perceive the benefits of behaviors that reduce the likelihood of that outcome. Previous research (Rubin et al., 2009) has shown that controlling for personal elements, namely perceptions, trust and anxiety is important: the “recommended changes were associated with perceptions that disease is severe, that the risk of catching it is high risk, that the outbreak will continue for a long time, that the authorities can be trusted, that good information has been provided, that people can control their risk of catching it, and that specific behaviors are effective in reducing the risk.” There is a fair amount of literature on risk communication and public trust (see e.g., Prati et al., 2011). It is known that social factors and social trust are crucial here (Phelan and Link, 1995; Kawachi et al., 1999; Hawe and Shiell, 2000; Subramanian, 2002; Kim et al., 2006). There is also research on risk perception and public trust (Weerd et al., 2011). We also have some incidental evidence on public safeguarding measures and social factors in some countries, including Slovakia (Perugini and Vladisavljevic, 2020), that shows that social and economic background (not just perceptions of the coronavirus threat) have an effect on actors’ behaviors–for example, socially and economically vulnerable actors with precarious jobs cannot afford to stop working or limit their work hours, especially in countries like Slovakia where state help and/or innovative work solutions are sluggish and inelastic. Actors who have continued to work may have very different attitudes concerning the perceived threat of the coronavirus.

The new Confidence in Safeguards scale (CSS) contains three dimensions (Confidence in Institutions, Confidence in Personal and Family Behaviors, Confidence in Others’ Behaviors), and the literature has already provided a rationale for distinguishing between cognitive versus affective risk appraisal in the adapted Perceived Risk of HIV Scale (PRHIV), see Oh et al. (2015). We therefore formulated three hypotheses on the relation between public trust and perceived threat of coronavirus:

(1) Confidence in behaviors of self/family will positively predict Fear of Contraction–the rationale is that respondents who adapt strict safeguarding measures are more anxious. On the other hand, respondents have more control over their own behavior and the behavior of their family members, and their fear of contraction could positively enhance their behavior and the family’s behavior, in turn increasing levels of confidence in self/family behaviors.
(2) Confidence in others’ behaviors will negatively predict both parts of Perceived Risk of Coronavirus–the rationale is that others’ behavior lies outside the respondent’s control, but it is also true that if others follow the safeguarding measures that could contribute to reducing the risk of infection.
(3) Confidence in institutions will negatively predict both parts of Perceived Risk of Coronavirus–the rational is practically the same as in the previous hypothesis: the institutional response is outside the respondent’s control and could have an impact on the spread of the coronavirus.

## Aim of the Study

The coronavirus pandemic is unprecedented. Therefore we have no hypotheses–this research is exploratory and the goal is to describe (rather than explain) some important patterns in perceptions and reasoning during this crisis. Our research goals were to: (1) develop a reliable instrument to investigate people’s confidence in the safeguarding measures (both institutional and behavioral); (2) adapt the instrument to measure perceived risk of coronavirus; and (3) to investigate the effect of public confidence in safeguarding measures designed to halt the spread of the coronavirus on perceived risk, controlling for related covariates.

## Methods

### Setting: Publicly Available Data on the Spread of COVID-19 in Slovakia in March 2020

First of all we present the situation in Slovakia in March 2020, providing information on the spread of the coronavirus, including the rate of testing. For the sake of comparison, data from neighboring countries (Czechia, Poland, Hungary, Austria) is presented as well. Secondly, we list the safeguarding measures implemented by the Slovak authorities during this period (March 2020).

**FIGURE 1:**
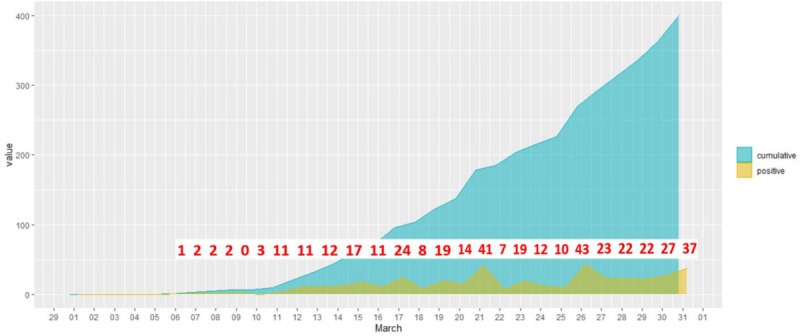
Daily increase in confirmed cases in Slovakia in March 2020.

In Slovakia the first positive case of COVID-19 was officially announced on 6 March 2020 (The National Health Information Centre, Extended Statistics, 2020). The daily increase in the numbers testing positive remained very low (under 5) until 11 March. Figure 1 presents the daily increase in confirmed cases in Slovakia in March 2020 (based on data from The National Health Information Centre, Extended Statistics, 2020). We can see that the cumulative growth curve is linear and does not show exponential growth: the daily increase in numbers is still relatively low (under 50). The total number of confirmed cases in Slovakia had reached 400 by 31 March.

We must also take into account the number of tests performed: the number of tests is relatively low, but Slovakia is capable of conducting the same number of tests on average as its neighbors (recalculated using a log scale per million inhabitants see Roser et al., 2020).

If we compare the number of confirmed cases in Slovakia in March to those in neighboring countries, the time series for Slovakia has an almost identical shape to that for Hungary, and similar to that for the Czechia and Poland. The time series for these countries differ markedly from Austria’s.

We can now turn our attention to the safeguarding measures adopted in Slovakia by the state authorities. On 9 March all schools and universities were closed, and on 10 March public events were banned. On 13 March all international passenger transport to and from Slovakia was canceled. On 16 March a state of emergency was declared, and all private businesses (except food shops, health and beauty retailers, pharmacies, and petrol stations) and state offices were closed. On 25 March the wearing of a mask covering the nose and mouth became compulsory in public. On the same day the Slovak parliament passed a “corona law” which permits (among other things) the government to use mobile location data to monitor user location.

We conclude this section by pointing out that Slovakia has a low number of confirmed COVID-19 cases and strict safeguarding measures in place, but this is not exceptional in Central Europe. We would like to stress that this conclusion has nothing to do with the epidemiological situation: we are simply reviewing the public and published resources that are widely available in Slovakia–our research design focuses on public trust and the perceived threat of coronavirus. The epidemiological situation (i.e., the population ratio of infected people) is unknown and may differ substantively from the number of confirmed cases.

### The Research Sample

The sample consisted of *N* = 565 respondents; 119 were males (21.1%) and 446 were females (78.9%). Mean age was 35.42 (SD = 13.11), range was 18–77 years. All the participants were Slovak citizens. Data were gathered via social networks between March 27, 2020 and March 31, 2020. We used convenience sampling. Data were collected in accordance with the ethical standards of the institutional and/or national research committee and in accordance with the 1964 Helsinki Declaration and its later amendments or comparable ethical standards.

### Measures

#### Perceived Risk of Coronavirus Scale (PRCS)

The PRHIV (Napper et al., 2012) has been adapted. The adaptation is similar to the adaptation for Ebola–the Perceived Vulnerability to Ebola risk scale (PVE; Kim et al., 2016). The scale originally contained 10 items but in the original study the authors excluded two of the items from the scale (Napper et al., 2012). The remaining items were then reformulated using a unified answer format which is more understandable and makes it easier and quicker for participants to complete. We also changed the name of the contagious disease from HIV to coronavirus. For the list of PRC items see Appendixes 1–3, Supplementary Materials. The psychometric analysis of the original scale was reproduced in this study, and modified slightly (see “Results” and Supplementary Materials, Appendixes 1–3), since the literature has already provided a rationale for distinguishing between cognitive versus affective risk appraisal, see Oh et al., 2015).

#### Confidence in Safeguards Scale (CSS)

The CSS contains ten items and was created for this research (see Appendix 2). It contains three dimensions: (1) Confidence in Institutions, (2) Confidence in Personal and Family Behaviors, and (3) Confidence in Others’ Behaviors. We decided to include these three dimensions so three aspects could be distinguished–confidence in personal behavior and family members’ behavior, which is at least partially within the respondent’s control; confidence in others’ behavior, which lies outside the respondent’s control; and confidence in institutional behavior, which also lies outside the respondent’s control but is represented differently. We deliberately avoided mentioning specific safeguarding behaviors (e.g., wearing masks, washing hands, social distancing, and staying at home): respondents could have very different opinions as to which behaviors they consider safe, and the aim was not to measure the prevalence of specific behaviors, but rather the overall confidence of respondents in any safeguarding behaviors: for example, item 4 (“My family members behave with adequate caution in regard to the spread of the coronavirus”) makes no reference to specific behaviors: it measures the respondent’s perception, not the occurrence of specific behaviors. We realize that respondents may have inconsistent opinions on safe behaviors in both directions (they might be confident the behaviors are safe for no objective reason, or they might consider even very precautionary behaviors as being insufficiently safe). Consequently this instrument is designed to measure confidence only. A psychometric analysis of this new scale was conducted as part of this study (see “Results” and Supplementary Materials, Appendixes 1–3).

#### Sociodemographic Data–Covariates

We have included the standard set of demographic variables (gender, age, education, size of residential site, number of household members, number of children aged under 18 in the household). Some of these could have a direct effect on perceived threat (women generally display more anxiety than men, see Remes et al., 2016; old people and people living in small villages or towns could be more vulnerable, see Garnier−Crussard et al., 2020; Ranscombe, 2020; Zhang and Schwartz, 2020). Over and above this standard set, two other specific covariates were included: (1) taking any prescribed medication (this factor could influence respondents’ perceptions and behavior), and (2) work attendance (respondents who go out to work could perceive the threat of coronavirus differently). Controlling for these covariates is important because otherwise some or all the structural regression coefficients could produce spurious results–false negatives or false positives (or both).

## Data Analysis

### Procedure

As far as the data are concerned, after presenting the descriptive statistics, we first investigate the psychometric properties of both scales. We replicate the item-response theory (IRT) approach used in the original PRHIV (Napper et al., 2012) and check its factor structure (the literature has already provided a rationale for distinguishing between cognitive versus affective risk appraisal, see Oh et al., 2015) and reliability. Non-parametric IRT kernel smoothing (Ramsay, 1991, 1997) is used to check the monotonicity of the expected item scores (EIS). We then propose some essential amendments based on the IRT re-analyses of our data. For all the IRT analyses, we use statistical program R, version 3. 6. 1 (R Core Team, 2019), packages “mirt” (Chalmers, 2012) for the IRT factor models and residuals analyses, and package “KernSmoothIRT” (Mazza et al., 2014) for the IRT kernel smoothing analysis. See Supplementary Materials, Appendixes 1–3 for all R codes.

The next step is the ESEM (exploratory structural equation modeling, see Asparouhov and Muthén, 2009; Marsh et al., 2011) factor analysis of the new CSS. The ESEM approach combines the confirmatory factor analytical approach (factor structure is specified in advance in target rotation) with the exploratory approach (in target rotation, some small cross-loadings on different factors are allowed, which is more realistic with regards to the data than setting them to zero). This approach is appropriate in our case because we have theoretically justified the factor structure of this instrument (confirmatory approach), but not yet tested it on the data (exploratory approach). Finally we fit the structural model where the CSS latent factors are predictors of the Perceived Risk of the Coronavirus Scale latent factors, controlling for the covariates: gender, age, education, size of residential site, number of household members, number of children under 18 in household, taking any prescribed medication, and work attendance. See Figure 1 for this structural model. We then check the fit of this model with the data (Comparative Fit Index CFI and Tucker-Lewis Index TLI should be >0.95 for excellent fit and >0.90 for acceptable fit, and RMSEA index should be <0.050 for excellent fit and <0.080 for acceptable fit, see Hu and Bentler, 1999; Kline, 2005). All ESEM models are fitted in the Mplus program, version 7.4 (Muthén and Muthén, 1998-2017). See Mplus codes in Supplementary Materials, Appendix 1–3.

## Results

### Descriptive Statistics

Descriptive statistics for both scales (and covariates) are presented in Table 1.

**TABLE 1:** Descriptive statistics of the PRC & CSS scales and covariates.

**Perceived Risk of Coronavirus Scale (PRCS)**							
**Item**	**Mean**	**SD**	**1**	**2**	**3**	**4**	**5**

cor01	2.45	1.02	0.20	0.31	0.34	0.12	0.03
cor02	3.38	1.21	0.08	0.18	0.24	0.29	0.21
cor03	2.96	1.31	0.15	0.25	0.23	0.21	0.16
cor04	2.99	1.35	0.15	0.27	0.21	0.18	0.19
cor05	3.83	1.21	0.06	0.09	0.23	0.22	0.40
cor06	2.97	1.25	0.13	0.25	0.26	0.22	0.14
cor07	2.46	1.10	0.20	0.37	0.26	0.11	0.06
cor08	2.37	1.21	0.29	0.30	0.21	0.13	0.07

**Confidence in Safeguards Scale (CSS)**							

Item	Mean	SD	1	2	3	4	5
pre01	4.26	0.85	0.01	0.03	0.11	0.38	0.46
pre02	4.53	0.64	0.01	0.01	0.04	0.34	0.60
pre03	3.95	0.92	0.02	0.06	0.15	0.50	0.27
pre04	4.39	0.77	0.01	0.02	0.08	0.37	0.52
pre05	2.32	0.97	0.22	0.36	0.32	0.08	0.02
pre06	3.63	0.97	0.03	0.09	0.26	0.45	0.17
pre07	2.81	1.07	0.12	0.27	0.35	0.20	0.06
pre08	3.89	0.94	0.02	0.03	0.27	0.38	0.30
pre09	2.67	1.02	0.14	0.26	0.44	0.11	0.05
pre10	3.31	0.95	0.04	0.15	0.35	0.39	0.07

**Covariates**							

	0	1	2	3	4	5	6
Gender	0.21	0.79	–	–	–	–	–
Education	0.01	0.30	0.69	–	–	–	–
Site	0.05	0.25	0.14	0.16	0.08	0.32	–
Medications	0.61	0.39	–	–	–	–	–
Work	0.90	0.10	–	–	–	–	–
Household	0.07	0.28	0.24	0.25	0.12	0.04	–
Children	0.63	0.22	0.12	0.03	–	–	–

*PRC & CSS scales: SD = standard deviation. Percentages of responses: 1 = strongly disagree, 2 = disagree, 3 = I don’t know, 4 = agree, 5 = strongly agree. Covariates percentages of responses: gender0 = males, gender1 = females. education0 = elementary, education1 = secondary school, education3 = university. site0 = under 500, site1 = 501-5000, site2 = 5001-20000, site3 = 20001-50000, site4 = 50001-100000, site5 = over 100000. medications0 = no, medications1 = yes. work0 = no, work1 = yes, household0 = single, household1 = 2, household2 = 3, household3 = 4, household4 = 5, household5 = 6 or more. children0 = 0, children1 = 1, children2 = 2, children3 = 3 or more.*

### Psychometric Analysis of the Perceived Risk of Coronavirus Scale (PRCS)

The final version of the original PRHIV (Napper et al., 2012) contains eight items. However, based on their original analyses of the ten items, the authors subsequently dropped two items with suboptimal psychometric properties. The authors checked the unidimensionality and local independence of this instrument and claimed it sufficiently unidimensional based on the RMSEA and GFI fit indices for the one-factor confirmatory factor analysis model (Napper et al., 2012). However, there are serious doubts about this claim: (1) the GFI index is very sensitive to sample size, and its use is discouraged in the relevant psychometric literature (Shevlin and Miles, 1998; Fan and Sivo, 2007); (2) the authors report that the value of RMSEA for the one-factor model is 0.087 and declare that supports use of this model, but this declaration directly contradicts the psychometric recommendations (RMSEA under 0.05 for excellent fit, under 0.08 for acceptable fit, see Bentler, 1990; Hu and Bentler, 1999; Kline, 2005; Chen et al., 2008). Moreover, the 90% confidence interval of the reported RMSEA of 0.087 is 0.078–0.095 (our own calculation based on χ^2^(35) = 242 and *N* = 785 reported by the authors) which is barely acceptable and so cannot be an indication of good fit.

Following the original procedure in Napper et al. (2012), the PRCS items were modeled using Samejima’s two-parameter polytomous graded response model (GRM) with marginal maximum likelihood estimation, using “mirt” package (Chalmers, 2012) in statistical program R, version 3. 6. 1. (R Core Team, 2019). We do not report the GFI; instead we report the RMSEA (with 90% CI) and SRMSR as recommended in the psychometric literature on goodness of fit for IRT models (Maydeau-Olivares, 2013). The one-factor model had an unacceptable RMSEA (0.116, 90% CI 0.101–0.132) and SRMSR (0.121). An inspection of the residuals (based on G2^∗^ statistics with better properties than a mere inspection of the residual correlations, see Chen and Thissen, 1997; Houts and Edwards, 2013) showed a distinctive pattern in the signed values of the Cramer phi indices, indicating a second factor (see Supplementary Materials, Table 1). Moreover our inspection of the EIS by means of non-parametric IRT kernel smoothing (Ramsay, 1991, 1997; Mazza et al., 2014) clearly indicated that item 4 was a problem (and that item 8, while less problematic, was suboptimal, see Supplementary Materials, Figure 1).

Since the literature has already provided a rationale for distinguishing between cognitive versus affective risk appraisal, see Oh et al. (2015), we tried to fit a two-factor EFA model, and the fit improved substantially (RMSEA = 0.048, 90% CI 0.021–0.078; SRMSR = 0.046). The subsequent inspection of the EIS based on two dimensions showed that the problems with items 4 and 8 had disappeared, see Supplementary Materials, Figure 2). The factor loadings of this two-factor model are presented in Table 2. Looking at the item content we can see that the items expressing affective content (e.g., worrying, feeling vulnerable) load on the first factor, and items expressing cognitive content (likelihood, chance, thinking) load on the second factor. The correlation between the latent factors is 0.362.

**FIGURE 2:**
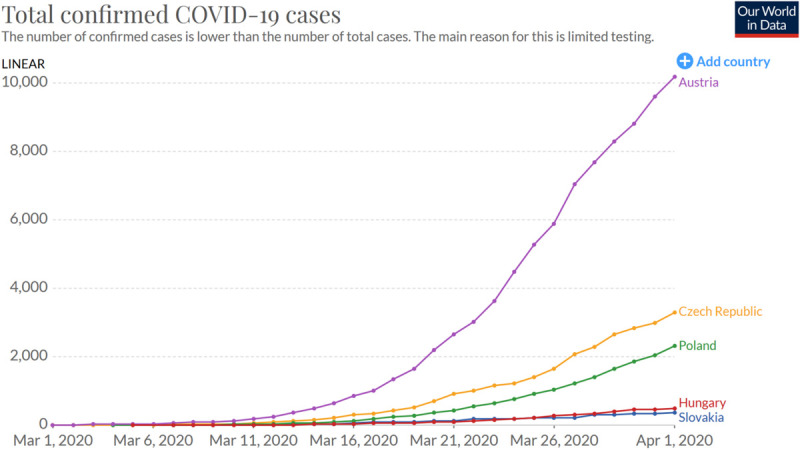
Number of total confirmed COVID-19 cases in five Central European countries (Roser et al., 2020).

**TABLE 2:** Factor loadings of two-factor model of the PRCS scale, IRT EFA analysis.

	**Item**	**Factor 1 (Fear)**	**Factor 2 (Likelihood)**
1.	I have a gut feeling I am likely to get infected with coronavirus.	0.090	**0.799**
2.	There is a chance, no matter how small, I could get coronavirus.	0.025	**0.499**
3.	I worry about getting infected with coronavirus.	**0.782**	0.195
4.	I find it hard to picture myself getting coronavirus.	**0.667**	−0.195
5.	I am sure I will NOT get infected with coronavirus.*	0.074	**0.606**
6.	I feel vulnerable to coronavirus infection.	**0.668**	0.230
7.	I think my chances of getting infected with coronavirus are large.	0.199	**0.703**
8.	I have often thought about getting coronavirus.	**0.665**	0.170

Factor loadings higher than 0.300 are presented in bold. *Item 5 was reverse scored.

Empirical IRT reliability, based on the weighted likelihood estimates of latent abilities, was 0.83 for the Fear dimension and 0.83 for the Likelihood dimension. Cronbach alpha was 0.75 for the total scale (eight items), 0.72 for the Fear dimension (four items), and 0.71 for the Likelihood dimension (four items). Therefore, we consider the PRCS to be a two-dimensional construct consisting of the Fear of Contraction subscale containing four items, and the Perceived Likelihood of Contraction subscale containing four items. Moreover, the literature has already provided a rationale for distinguishing between cognitive versus affective risk appraisal, which fits nicely with these results (see Oh et al., 2015).

### Psychometric Analysis of the Confidence in Safeguards Scale (CSS)

**FIGURE 3:**
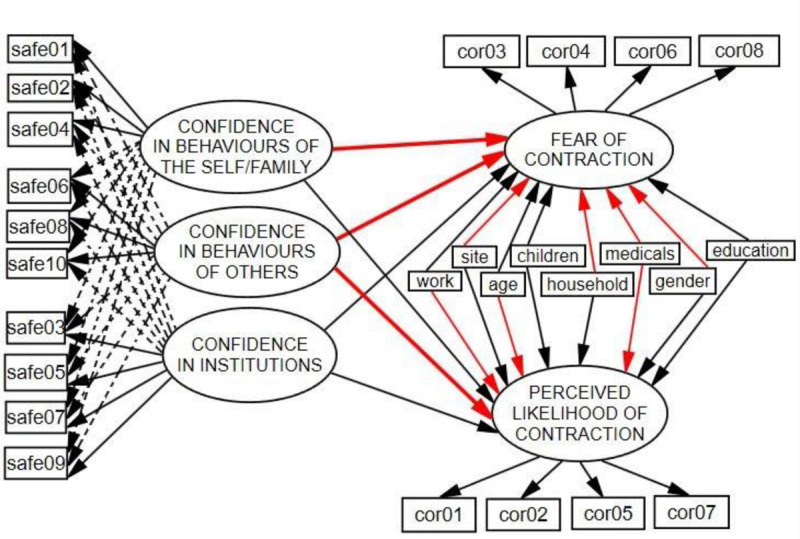
Final ESEM structural model. Significant regression coefficients are indicated in red. Dashed lines are cross-loadings set to minimum in target rotation.

The CSS was created as a ten-item three-dimensional instrument, containing these three dimensions: Confidence in Institutions, Confidence in Personal and Family Behaviors, and Confidence in Others’ Behaviors. The ESEM model was fitted using the WLSMV estimator (weighted least squares mean and variance adjusted method, which performs well with ordinal items, see Beauducel and Herzberg, 2006; Bandalos, 2014) and target rotation specifying the theoretically justified loadings, but allowing some small cross-loadings at the same time. See Figure 3, the CSS model.

The three-factor ESEM model has a good fit with the data: χ^2^(18) = 70.88, CFI = 0.97, TLI = 0.93, RMSEA = 0.072 (90% CI 0.063–0.080). Factor loadings are shown in Table 3.

**TABLE 3:** Factor loadings of three-factor ESEM model of the CSS scale, target rotation.

	**Item**	**Factor 1 (Self/Family)**	**Factor 2 (Others)**	**Factor 3 (Institutions)**
1.	I have enough information about the spread of the coronavirus.	**0.409**	0.048	0.145
2.	I behave with adequate caution in regard to the spread of the coronavirus.	**0.993**	−0.162	−0.031
3.	The authorities are taking adequate safeguards against the spread of the coronavirus.	0.103	0.194	**0.382**
4.	My family members behave with adequate caution in regard to the spread of the coronavirus.	**0.464**	0.322	−0.021
5.	Medical facilities are prepared for the spread of the coronavirus.	0.046	−0.098	**0.682**
6.	My neighbors and the people I meet behave with adequate caution in regard to the spread of the coronavirus.	0.007	**0.769**	0.019
7.	Shops, pharmacies, and drugstores are prepared for the spread of the coronavirus.	−0.051	−0.034	**0.775**
8.	My fellow workers behave with adequate caution in regard to the spread of the coronavirus.	0.128	**0.583**	−0.016
9.	Banks and financial services are prepared for the spread of the coronavirus.	−0.011	0.095	**0.539**
10.	Overall, people in my country behave with adequate caution in regard to the spread of the coronavirus.	−0.029	**0.454**	0.209

*Factor loadings specified in target rotation are presented in bold.*

We can see that both the fit indices and factor loadings support the three-dimensional model of the CSS scale: items expressing confidence in personal and family behaviors predominantly load on the first factor, items assigned to others’ behaviors load mostly on the second factor, and items expressing confidence in institutions load mostly on the third factor. The correlations among latent factors are 0.440 (confidence in personal/family behaviors with confidence in others’ behaviors), 0.262 (confidence in personal/family behaviors with confidence in institutions), and 0.554 (confidence in others’ behaviors with confidence in institutions). All are statistically significant at 0.001. Cronbach alpha for the total scale was 0.75, and for the subscales 0.70 (Confidence in Personal and Family Behaviors), 0.73 (Confidence in Others’ Behaviors), and 0.72 (Confidence in Institutions).

### The Final ESEM Structural Model

The final ESEM structural model is presented in Figure 3 (error variances, disturbances, and covariances are omitted). This model has a good fit with the data: χ^2^(239) = 508.88, CFI = 0.94, TLI = 0.93, RMSEA = 0.045 (90% CI 0.039–0.050). Regression weights are presented in Table 4.

**TABLE 4:** Regression weights from the final ESEM structural model.

**Regression path**	**β (SE)**	***p*-value**	**SD value**
FEAR OF CONTRACTION ∼ confidence in self/family	**0.138 (0.06)**	0.013	0.153
FEAR OF CONTRACTION ∼ confidence in others	−**0.270 (0.07)**	<0.001	–0.299
FEAR OF CONTRACTION ∼ confidence in institutions	−0.058 (0.06)	0.330	–0.064
LIKELIHOOD OF CONTRACTION ∼ confidence in self/family	0.028 (0.05)	0.558	0.035
LIKELIHOOD OF CONTRACTION ∼ confidence in others	−**0.159 (0.05)**	0.003	–0.201
LIKELIHOOD OF CONTRACTION ∼ confidence in institutions	−0.052 (0.05)	0.289	–0.065
FEAR OF CONTRACTION ∼ age	0.001 (0.01)	0.808	0.012
FEAR OF CONTRACTION ∼ gender	**0.288 (0.10)**	0.004	0.319
FEAR OF CONTRACTION ∼ work	−0.030 (0.14)	0.833	–0.033
FEAR OF CONTRACTION ∼ medications	**0.439 (0.09)**	<0.001	0.486
FEAR OF CONTRACTION ∼ education	−0.168 (0.09)	0.072	–0.086
FEAR OF CONTRACTION ∼ site	−**0.052 (0.02)**	0.032	–0.099
FEAR OF CONTRACTION ∼ household	−**0.111 (0.04)**	0.008	–0.155
FEAR OF CONTRACTION ∼ children	0.114 (0.06)	0.059	0.104
LIKELIHOOD OF CONTRACTION ∼ age	−**0.007 (0.01)**	0.032	–0.115
LIKELIHOOD OF CONTRACTION ∼ gender	−0.067 (0.09)	0.449	–0.084
LIKELIHOOD OF CONTRACTION ∼ work	**0.310 (0.13)**	0.017	0.392
LIKELIHOOD OF CONTRACTION ∼ medications	**0.181 (0.08)**	0.022	0.228
LIKELIHOOD OF CONTRACTION ∼ education	0.116 (0.09)	0.190	0.068
LIKELIHOOD OF CONTRACTION ∼ site	0.032 (0.02)	0.157	0.070
LIKELIHOOD OF CONTRACTION ∼ household	−0.075 (0.04)	0.057	–0.119
LIKELIHOOD OF CONTRACTION ∼ children	0.071 (0.06)	0.226	0.073

*Bold = Statistically significant coefficients are presented in bold. SD value = standardized value.*

## Discussion

In this exploratory research study we developed an instrument to investigate people’s confidence in safeguarding measures (CSS), adapted the instrument measuring the perceived risk of coronavirus (PRCS), and explored the effect of public confidence in safeguarding measures designed to prevent the spread of the coronavirus on perceived risk, controlling for related covariates (gender, age, education, size of site, number of household members, number of children under 18 in household, taking any prescribed medication, and work attendance).

The three-factor ESEM model for the CSS had a good fit with the data, confirming a three-dimensional structure: Confidence in Institutions, Confidence in Personal/Family Behaviors, and Confidence in Others’ Behaviors. In addition the correlations among the three latent factors were statistically significant. The Cronbach alpha for the scale was adequate at 0.75. The CSS showed good psychometric properties and the expected factor structure and consequently the CSS can be used to reliably measure confidence in safeguards to prevent the spread of Coronavirus at three levels: personal and family, other people, and institutions. Admittedly, the three items of the “Self/Family” factor are mixed–two of them are formulated such that they express the person’s own behavior, and one item is formulated such that it expresses the behavior of family members. This is probably the reason this “family” item has a moderate cross-loading on the “Others” factor. Item 1 (“I have enough information.”) is passive, and could be amended to “I try to get up-to-date information about the spread of the coronavirus.” In the same vein, Item 4 could be amended to “As a family, we all behave with adequate caution.” This formulation refers to both the person’s own behavior and the behavior of family members. We recognize that including the family item along with items referring to the person’s own behavior introduces an element of confounding here, but we assume that many respondents will have a tightly knit family life (reinforced during the pandemic, for good or for worse), and having only individualistic and institutional items (i.e., omitting the family item) would be too restrictive. On the one hand, one can far more easily avoid interactions with non-family others than interactions with family members, and on the other hand, people have more influence on the behavior of family members than on the behavior of non-family others. At the height of the pandemic, families were often the centre of social life, and arguably “family members” and non-family others are not equivalents. Therefore merging the self and family precautions is to an important extent necessary: removing the family item from the “self” factor and including it in the “others” factor would probably distort the situation. In future research it would probably be worth considering a four-dimensional construct: Self, Family, Others, and Institutions–but adding the family item to the others factor would probably be too restrictive.

The PRCS was adapted from the PRHIV (Napper et al., 2012). Although Napper et al. (2012) claim the scale is unidimensional, their reported results (RMSEA value and confidence interval) do not support this. Similarly, our PRCS results show that the scale has two factors, not one. The two factors represent Fear of Contraction (e.g., worrying, feeling vulnerable, picturing self, and recurrent thoughts) and Perceived Likelihood of Contraction (e.g., likelihood, chance, thinking, and gut feeling) which correlated together at the level of 0.362. Concerning reliability, the scale showed good reliability coefficients (Cronbach alpha was 0.75 for the total scale, for Fear of Contraction it was 0.72, and for Perceived Likelihood of Contraction it was 0.71. This two-dimensional structure makes sense in interpretational terms: we can easily assume different patterns of responses–one respondent could perceive a high risk cognitively, but still express either high affective concern or low affective concern; while another respondent could perceive a low risk cognitively, but still feel either high affective concern, or low affective concern. Without considering these two different patterns, we could obtain distorted results.

Now we will discuss the hypotheses we formulated in the introduction:

(1) Confidence in behaviors of self/family will positively predict Fear of Contraction: this hypothesis has been confirmed. Confidence in personal and family behaviors predicts fear of contraction and is statistically significant and positive, but it does not predict the perceived likelihood of contraction. Respondents who are more confident about their own and their family’s precautionary behaviors are more scared of becoming infected: they affectively perceive the coronavirus to be a threat, but not cognitively. It could be that some form of circular causality is at work here: their fear of contracting the coronavirus could in turn boost their precautionary behaviors, but not enough to alleviate their cognitive perception of this threat. It is likely that worrying about contagion motivates more precautionary behaviors. From a clinical psychology perspective it makes perfect sense that this does not alleviate worries about contagion because the precautions act as “safety behaviors.” These offer short-term relief from worry but in the long run confirm and thereby maintain the worry beliefs. This is inflated even more if confidence in others’ behaviors is low, as we cannot control others’ behaviors to the extent we can control our own behavior. This is in line with models of generalized anxiety disorder (Lissek et al., 2014; Stein and Sareen, 2015), because it shows so clearly why the processes underlying generalized anxiety disorder can be adaptive depending on the context. These results suggest that this mechanism is probably one that policy should exploit.
(2) Confidence in others’ behaviors will negatively predict both parts of Perceived Risk of Coronavirus: this hypothesis has been confirmed as well because confidence in others’ behaviors predicts both fear of contraction and perceived likelihood of contraction and is statistically significant and negative: the more confidence people have in the precautionary behaviors of others, the less of a threat they consider the spread of the coronavirus, both affectively and cognitively. People who worry about becoming infected will escalate their safeguarding behaviors. At the same time, people who think others are not doing the same as them will become even more worried. A side effect of this is that people will also perceive that the likelihood of contraction is higher if others are not following the safeguards (after all, “all the others” constitutes a much bigger proportion of the population endangering them than just “me and my family”). We should also clarify that the PCRS “cognitive” factor (perceived likelihood of contraction) assesses the perceived likelihood of contracting the coronavirus, and not necessarily cognitive appraisals of the fear of the negative consequences of becoming infected (this comes under the “affective” factor, fear of contraction). People in Slovakia who have confidence in others’ safeguarding behaviors (but not in their own and their family’s safeguarding behaviors) consider the coronavirus to be less of a threat. Respondents probably also take into account the fact that social factors are predominantly behind the spread of the coronavirus. This trust in social solidarity is very promising and should be reinforced as much as possible, providing it is strongly connected to the actual mechanisms of coronavirus infection–the best protection is to monitor and avoid people who ignore safeguarding measures. The fact that confidence in others’ behavior is more predictive than confidence in self/family behavior seems to be related to the way the contagion spreads and the anti-contagion measures.
(3) Confidence in institutions will negatively predict both parts of Perceived Risk of Coronavirus–this hypothesis has not been confirmed. In general the Slovak respondents display low confidence in institutions and their preparedness for the coronavirus pandemic (see Table 1), and there is no statistically significant relationship between confidence in institutions and perceived threat of the coronavirus.

As far as the covariates are concerned, there is no statistically significant relationship between education level and number of children in the household and Fear of Contraction or Perceived Likelihood of Contraction. Taking medication significantly and positively predicts both fear of contraction and perceived likelihood of contraction: people taking medication think the spread of coronavirus is more of a threat, which comes as no surprise. Respondents who go out to work (versus those staying at home) show a significant and positive relationship with the perceived likelihood of contraction (cognitive awareness of being at a higher risk), but they do not show a significant relation to fear of contraction. Women (compared with men) display significantly more anxiety (there is a statistically significant and positive relation with fear of contraction), but there is no significant gender difference in relation to the perceived likelihood of contraction, which is again in accordance with previous research (Remes et al., 2016). Respondents living in small sites display more fear of contraction, but less perceived likelihood of contraction (or, to put it the other way around, respondents living in larger sites perceive the coronavirus as less threatening). This finding might seem to run counter to the facts because almost all the main confirmed outbreaks of coronavirus infection in Slovakia and in other countries are located in large cities, but incidental evidence shows that rural areas may be uniquely vulnerable due to the older age structure of many rural communities, the higher prevalence of chronic illnesses, and relative lack of health care facilities and services (Ranscombe, 2020; Zhang and Schwartz, 2020). The same strange pattern of reasoning is valid for age: older respondents think (cognitively) that they are less likely to be infected even though statistically older people are at a higher risk of becoming seriously ill with the coronavirus (Garnier−Crussard et al., 2020). And finally, the more members a household has, the less the coronavirus is affectively perceived to be a threat. This is probably the dual effect of consolation from family members on the one hand, and the psychological effect of loneliness on the other.

### Limitations of the Study

The first limitation consists in the fact that the sample is not representative for the Slovak population: educated respondents, women, and inhabitants of larger sites are overrepresented. Therefore, our results cannot be generalized to the Slovak population.

The second limitation is that we had neither the opportunity nor the time to adopt the proper procedures for validating and piloting our measures: according to psychometric standards, we should have collected the first set of data, then conduct detailed psychometric analyses, and only after inspecting the psychometric properties of our instruments and making the necessary adjustments, should we have begun the second wave of data collection. However, it would be extremely difficult to follow this procedure in the ongoing coronavirus crisis: it would require weeks and the situation is changing so rapidly that the prospective piloting sample could end up measuring very different perceptions from the reference sample collected after several weeks. We therefore decided to collect the data at the end of March 2020 with the aim of obtaining explorative insights instead of developing a more accurate instrument.

There is one more limitation: we cannot offer an adequate explanation for our outcomes–we lack background information about the respondents’ psychological characteristics, social background and networks, biographical histories, and so on. We are therefore unable to explain the significant variability and patterns observed in their responses. Further research is required to achieve this. This could be done using comparative research to replicate the data collection using the same instruments in different countries as soon as possible and comparing the results.

## Conclusion

Social trust (Subramanian, 2002; Kim et al., 2006) and other social conditions (Phelan and Link, 1995; Kawachi et al., 1999; Hawe and Shiell, 2000) are very important factors that strongly affect behaviors during any public health emergency, and the current coronavirus pandemic crisis is no exception. Our research indicates that Slovak respondents with higher levels of confidence in others’ behaviors perceived the spread of the coronavirus to be less threatening, both cognitively (perceived likelihood of contraction) and affectively (fear of contraction). Confidence in institutions and confidence in personal and family behaviors had no such effect. This finding should be considered when formulating public health policy and emergency communication to boost and enhance this effect–confidence in others’ behaviors could promote responsible attitudes: if others stick to the safeguarding measures, actors will consider their own behavior to be meaningful, in accordance with the social situation and their social reputation. However, confidence in others’ behaviors might reduce risk perception, and when trust in others’ behaviors is too high, actors may reconsider adhering to the safeguarding measures. The impact of social pressure and social reputation implied by the conformist adherence to the safeguarding measures will probably prevail: for example, recent results from Japan (Nakayachi et al., 2020) show that people conformed to social norms by wearing masks and at the same time felt relief from anxiety when wearing masks. Our findings (and other evidence) suggest that decision makers responsible for public health should consider social motivations when implementing public strategies to combat the COVID-19 pandemic.

## Data Availability Statement

The raw data supporting the conclusions of this article will be made available by the authors, without undue reservation.

## Ethics Statement

The studies involving human participants were reviewed and approved by Ethical Committee of Faculty of Social and Economic Sciences of Comenius University in Bratislava in Slovakia. The patients/participants provided their written informed consent to participate in this study.

## Author Contributions

Both authors designed the research, collected the data, wrote the manuscript, interpreted the results, revised the manuscript, and read and approved the final manuscript. MK performed the statistical analysis.

## Conflict of Interest

The authors declare that the research was conducted in the absence of any commercial or financial relationships that could be construed as a potential conflict of interest.
