# Supplementary material for: Perceived Threat of the Coronavirus and the Role of Trust in Safeguards: A Case Study in Slovakia
Source: Front Psychol. 2020 Nov 30;11:554160. doi: 10.3389/fpsyg.2020.554160 (PMC7733986; doi:10.3389/fpsyg.2020.554160)
Supplement: Supplementary file 1 [file Presentation_1.pdf]

Supplementary Materials

Appendix 1

The Perceived Coronavirus Risk scale (adapted from Napper, Fisher, & Reynolds, 2012)

Please read each sentence carefully before answering. Using the five-options answer below each sentence, select the appropriate number according to what is true for you.

	Item	1 Strongly disagree	2 Disagree	3 I do not know	4 Agree	5 Strongly agree
1.	I have a gut feeling I am likely to get infected with coronavirus.					
2.	There is a chance, no matter how small, I could get coronavirus.					
3.	I worry about getting infected with coronavirus.					
4.	I find it hard to picture myself getting coronavirus.					
5.	I am sure I will <u>NOT</u> get infected with coronavirus. *					
6.	I feel vulnerable to coronavirus infection.					
7.	I think my chances of getting infected with coronavirus are large.					
8.	I have often thought about getting coronavirus.					

* Reverse scored

	Položka	1 Vôbec nesúhlasím	2 Nesúhlasím	3 Neviem	4 Súhlasím	5 Úplne súhlasím
1.	Mám tušenie, že sa pravdepodobne nakazím koronavírusom.					
2.	Je tu nejaká malá šanca, že sa nakazím koronavírusom.					
3.	Mám obavy, že sa nakazím koronavírusom.					
4.	Je pre mňa ťažké predstaviť si, že sa nakazím koronavírusom.					
5.	Som si istý/á, že sa NENAKAZÍM koronavírusom. *					
6.	Cítim sa zraniteľný/á voči koronavírusu.					
7.	Moja šanca nakaziť sa koronavírusom je veľká.					
8.	Často myslím na nakazenie sa koronovírusom.					

* Reverzné skórovanie

Appendix 2

The Confidence in Coronavirus Safeguards scale

Please read each sentence carefully before answering. Using the five-options answer below each sentence, select the appropriate number according to what is true for you.

	Item	1 Strongly disagree	2 Disagree	3 I do not know	4 Agree	5 Strongly agree
1.	I have enough information about the spread of the coronavirus. [Original]<br>I try to get up- to- date information about the spread of the coronavirus. [Revised]					
2.	I behave with adequate caution in regard to the spread of the coronavirus.					

3.	The authorities are taking adequate safeguards against the spread of the coronavirus.					
4.	My family members behave with adequate caution in regard to the spread of the coronavirus. [Original]<br>As a family, we all behave with adequate caution in regard to the spread of the coronavirus. [Revised]					
5.	Medical facilities are prepared for the spread of the coronavirus.					
6.	My neighbours and the people I meet behave with adequate caution in regard to the spread of the coronavirus.					
7.	Shops, pharmacies, and drugstores are prepared for the spread of the coronavirus.					
8.	My fellow workers behave with adequate caution in regard to the spread of the coronavirus.					
9.	Banks and financial services are prepared for the spread of the coronavirus.					
10.	Overall, people in **my country*** behave with adequate caution in regard to the spread of the coronavirus.					

* replace with the name

	Položka	1 Vôbec nesúhlasím	2 Nesúhlasím	3 Neviem	4 Súhlasím	5 Úplne súhlasím
1.	Mám dostatočné množstvo informácií o šírení koronavírusu. [Originál]<br>Snažím sa získavať najnovšie informácie o šírení koronavírusu. [Revidované]					
2.	Správam sa dostatočne opatrne vzhľadom na šírenie koronavírusu.					
3.	Štátne orgány prijali dostatočné opatrenia proti šíreniu koronavírusu.					
4.	Členovia mojej rodiny sa správajú dostatočne opatrne vzhľadom na šírenie koronavírusu. [Originál]<br>Ako rodina sa všetci správame dostatočne opatrne vzhľadom na šírenie koronavírusu. [Revidované]					
5.	Zdravotnícke zariadenia sú pripravené na šírenie koronavírusu.					
6.	Moji susedia a ľudia, ktorých stretávam, sa správajú dostatočne opatrne vzhľadom na šírenie koronavírusu.					
7.	Obchody, lekárne a drogérie sú pripravené na šírenie koronavírusu.					
8.	Moji kolegovia a kolegyne z práce sa správajú dostatočne opatrne vzhľadom na šírenie koronavírusu.					
9.	Banky a finančné služby sú pripravené na šírenie koronavírusu.					
10.	Ľudia na Slovensku sa celkovo správajú dostatočne opatrne vzhľadom na šírenie koronavírusu.					

Appendix 3

R codes and Mplus code

Part 1: R code for IRT models

```
library(mirt)

library(Hmisc)

data = spss.get("C:/R/coronavirus.sav") # creating the dataframe "data"

dat = subset(data,select=c(10:17)) # extracting the dataframe "dat" with 8 coronavirus items

m1 = mirt(dat,1,itemtype="graded") # fitting the unidimensional model

summary(m1) # displaying factor loadings for the unidimensional model

M2(m1, type="C2") # displaying fit indices for the unidimensional model

residuals(m1, type="LDG2") # displaying residuals' G2 statistics (Table T1 below)

m2 = mirt(dat,2,itemtype="graded") # fitting the two-dimensional model

summary(m2) # displaying factor loadings for the unidimensional model (Table 2 in text)

M2(m2, type="C2") # displaying fit indices for the two-dimensional model
```

Part 2: R code for non-parametric IRT kernel smoothing

```
library(KernSmoothIRT)

ks = ksIRT(dat, rep(1, ncol(dat)), 1) # create IRT kernel smoothing input (8 items)

plot(ks, item=c(1:8),plottype="EIS") # plot 8 expected item scores (Figure F1 below)

dim1 = subset(dat,select=c(3,4,6,8)) # extracting "dim1" with 4 coronavirus items

ks1 = ksIRT(dim1, rep(1, ncol(dim1)), 1) # create IRT kernel smoothing input (4 items)

plot(ks1, item=c(2,4),plottype="EIS") # plot 2 expected item scores (Figure F2 below)
```

Part 3: Mplus code for the ESEM model of The Confidence in Coronavirus Safeguards scale

```
TITLE: ESEM model CSS in Mplus
    DATA: FILE = "C:/R/coronavirus.dat";
    VARIABLE:
    NAMES = age edu sex site medicals work household children
    cor01 cor02 cor03 cor04 cor05 cor06 cor07 cor08
    pre01 pre02 pre03 pre04 pre05 pre06 pre07 pre08 pre09 pre10;
    usevar = pre01 pre02 pre03 pre04 pre05 pre06 pre07 pre08
    pre09 pre10;
    categorical = pre01 pre02 pre03 pre04 pre05 pre06 pre07 pre08
    pre09 pre10;
    Analysis: ESTIMATOR = WLSMV;
```

```
Rotation = TARGET;
Model:
PRE1 BY pre01 pre02 pre03~0 pre04 pre05~0 pre06~0 pre07~0
pre08~0 pre09~0 pre10~0(*1);
PRE2 BY pre01~0 pre02~0 pre03~0 pre04~0 pre05~0 pre06 pre07~0
pre08 pre09~0 pre10(*1);
PRE3 BY pre01~0 pre02~0 pre03 pre04~0 pre05 pre06~0 pre07
pre08~0 pre09 pre10~0(*1);
OUTPUT: STDYX;
```

Part 4: Mplus code for the final ESEM structural model

```
TITLE: ESEM model final in Mplus
DATA: FILE = "C:/R/coronavirus.dat";
VARIABLE:
NAMES = age edu sex site medicals work household children
cor01 cor02 cor03 cor04 cor05 cor06 cor07 cor08
pre01 pre02 pre03 pre04 pre05 pre06 pre07 pre08 pre09 pre10;
usevar = work medicals household children age sex site edu
cor01 cor02 cor03 cor04 cor05 cor06 cor07 cor08
pre01 pre02 pre03 pre04 pre05 pre06 pre07 pre08 pre09 pre10;
categorical = cor01 cor02 cor03 cor04 cor05 cor06 cor07 cor08
pre01 pre02 pre03 pre04 pre05 pre06 pre07 pre08 pre09 pre10;
Analysis: ESTIMATOR = WLSMV;
Rotation = TARGET;
Model:
PRE1 BY pre01 pre02 pre03~0 pre04 pre05~0 pre06~0 pre07~0
pre08~0 pre09~0 pre10~0(*1);
PRE2 BY pre01~0 pre02~0 pre03~0 pre04~0 pre05~0 pre06 pre07~0
pre08 pre09~0 pre10(*1);
PRE3 BY pre01~0 pre02~0 pre03 pre04~0 pre05 pre06~0 pre07
pre08~0 pre09 pre10~0(*1);
COR1 BY cor01 cor02 cor05 cor07;
COR2 BY cor03 cor04 cor06 cor08;
COR1-COR2 ON PRE1-PRE3;
COR1-COR2 ON age;
COR1-COR2 ON sex;
COR1-COR2 ON site;
COR1-COR2 ON work;
COR1-COR2 ON medicals;
COR1-COR2 ON household;
COR1-COR2 ON children;
COR1-COR2 ON edu;
OUTPUT: STDYX STDY;
```

Table T1

The Perceived Coronavirus Risk scale, standardized residuals – signed Cramér phi version

	cor02	cor03	cor04	cor05	cor06	cor07	cor08
cor01	0.226	-0.163	-0.167	0.174	-0.155	0.198	-0.115
cor02		-0.134	-0.156	0.149	-0.151	-0.176	-0.140
cor03			0.213	-0.172	0.158	-0.146	0.106
cor04				-0.198	0.156	-0.127	0.122
cor05					-0.132	0.127	-0.136
cor06						-0.150	-0.098
cor07							-0.109

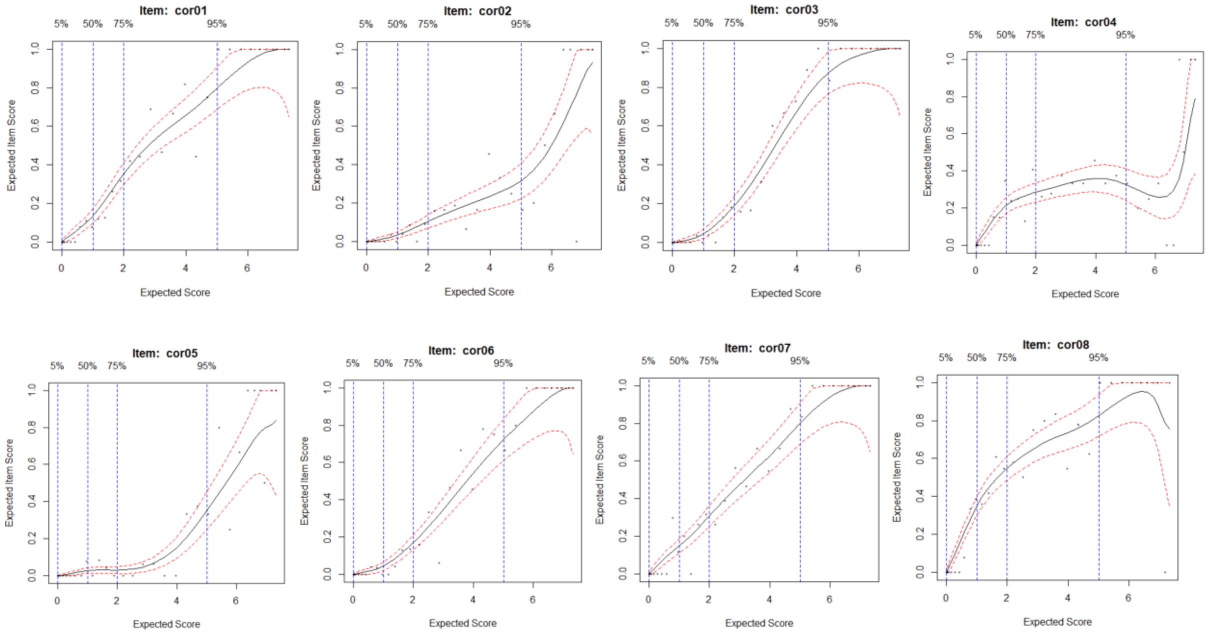

Figure F1. Expected Item Scores of items of the PRC scale (unidimensional).

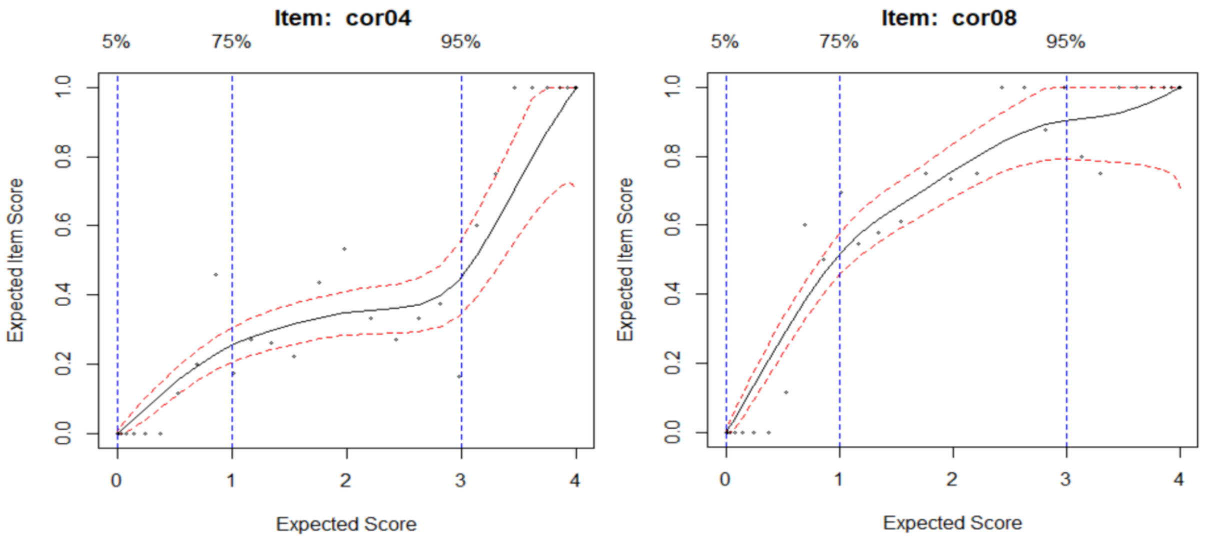

Figure F2. Expected Item Scores of 2 items of the PRC scale (two-dimensional).
